# Effects of Low Vitamin C Intake on Fertility Parameters and Pregnancy Outcomes in Guinea Pigs

**DOI:** 10.3390/nu15194107

**Published:** 2023-09-22

**Authors:** Sharna J. Coker, Rebecca M. Dyson, Carlos C. Smith-Díaz, Margreet C. M. Vissers, Mary J. Berry

**Affiliations:** 1 Perinatal and Developmental Physiology Group, Department of Paediatrics and Child Health, University of Otago, Wellington 6242, New Zealand; sharna.coker@postgrad.otago.ac.nz (S.J.C.); becs.dyson@otago.ac.nz (R.M.D.); 2 Mātai Hāora—Centre for Redox Biology and Medicine, Department of Pathology and Biomedical Science, University of Otago, Christchurch 8140, New Zealand; carlos.smith-diaz@postgrad.otago.ac.nz

**Keywords:** vitamin C (ascorbate, ascorbic acid), fertility, reproduction, preconception, pregnancy, foetus, neonate, guinea pig

## Abstract

Identifying how specific nutrients can impact fertility, pregnancy, and neonatal outcomes will yield important insights into the biological mechanisms linking diet and reproductive health. Our study investigates how dietary vitamin C intake affects various fertility parameters and pregnancy and neonatal outcomes in the guinea pig, a natural model of vitamin C dependency. Dunkin Hartley guinea pigs were fed an optimal (900 mg/kg feed) or low (100 mg/kg feed) vitamin C diet ad libitum for at least three weeks prior to mating and throughout pregnancy. We found that animals receiving the low vitamin C diet had an increased number of unsuccessful matings, a higher incidence of foetal reabsorption, and, among pregnancies resulting in delivery at term, produced fewer offspring. Neonates from mothers on the low vitamin C diet had significantly decreased plasma vitamin C concentrations at birth and exhibited mild growth impairments in a sex-dependent manner. We conclude that a diet low of vitamin C induces a state of subfertility, reduces overall fecundity, and adversely impacts both pregnancy outcomes and growth in the offspring. Our study provides an essential foundation for future investigations to determine whether these findings translate to humans. If so, they could have important clinical implications for assisted reproductive technologies and nutritional recommendations for couples trying to conceive, pregnant women, and breastfeeding mothers.

## 1. Introduction

Vitamin C is an essential nutrient sourced exclusively from the diet in humans and only a few other species, including guinea pigs, due to a non-functioning gulonolactone oxidase enzyme [1]. The roles of vitamin C are diverse and essential for maintaining the physiological function of all tissues, including the reproductive organs. This vital nutrient acts as a powerful antioxidant and co-factor for a plethora of different enzymes involved in hormone production, collagen synthesis, and epigenetic regulation.

Ovarian vitamin C concentrations increase around the time of ovulation. This has been observed in several species including guinea pigs [2], rats [3], and cattle [4]. Similarly in women, a change in the retention vs. urinary excretion of vitamin C occurs during the mid-phase of the menstrual cycle [5,6,7]. When dietary intake is adequate, the urinary excretion of vitamin C decreases just before ovulation [6,7]. This pattern reflects an increased demand for and uptake of vitamin C by the ovary to facilitate optimal ovulation and uterine function [8,9,10]. One of the principal roles of vitamin C in the ovary is to stimulate the production of luteal hormones, like progesterone, which prepare the endometrium for embryonic implantation and the maintenance of pregnancy [11,12,13]. In addition to its role in hormone production, vitamin C regulates collagen hydroxylation, a crucial process for ovarian functions like follicle growth, formation of the corpus luteum, and the repair of the ruptured follicle following ovulation [8,9,10]. Historical studies on severe vitamin C deficiency have documented ‘loss of the oestrous rhythm’ in guinea pigs [14] and hypermenorrhoea in women with scurvy, likely a combined effect of bleeding diathesis and hormonal changes such as lowered progesterone [15]. Based on the existing literature, inadequate vitamin C intake might disrupt the regulation of oestrous and menstrual cycles, impair ovarian and uterine function, and negatively impact female fertility.

Vitamin C also has the potential to impact male fertility parameters. The testes are a site of high vitamin C accumulation and turnover [16] and insufficient vitamin C may lead to a state of oxidative stress within the testes, which can impair sperm function and cause significant sperm loss [17,18,19]. Vitamin C supplementation has been shown to benefit male reproductive health in the context of specific fertility issues such as oligospermia [20]. Whether paternal vitamin C supplementation can improve spontaneous pregnancy rates and ongoing pregnancy outcomes for all couples trying to conceive remains unclear.

During normal gestation, vitamin C is preferentially transported across the placenta from mother to foetus [21,22] which suggests that the nutrient is critical for normal foetal development. However, this preferential transport into the foetus does not fully compensate for grossly inadequate maternal intake. In both humans and guinea pigs, low maternal vitamin C status during pregnancy also corresponds to low vitamin C status in the neonate [21,22,23]. Additionally, low vitamin C status in pregnant women has been associated with pregnancy complications such as an increased risk of preeclampsia [24,25], preterm delivery [26], and low birth weight infants [27,28]. Vitamin C consumption during pregnancy has also been reported to be positively correlated with infant growth up to 6 months of age [29]. Maternal vitamin C supplementation to improve these outcomes has produced inconsistent results [30,31,32,33]. Specific subgroups such as smokers, diabetics, and individuals with limited food variety, i.e., those individuals most at risk of vitamin C deficiency, are often excluded from or not discussed in relevant studies, highlighting a clear research gap that needs to be addressed.

The relationship between vitamin C, fertility, pregnancy, and neonatal outcomes is still controversial due to limited clinical evidence and inconsistent findings. When considering the impact of diet on reproductive health in humans, confounding variables such as socioeconomic status, possible comorbidities, geographical and seasonal produce availability, and varying food storage and preparation techniques make it difficult to establish causality between a given nutritional insult and resulting fertility issues or pregnancy complications. Well-designed animal studies allow us to overcome these limitations and study controlled dietary manipulations, such as low vitamin C intake, in vivo.

In this study, we investigated how dietary vitamin C intake affects various fertility parameters and pregnancy and neonatal outcomes in a clinically relevant guinea pig model. We report that a diet low in vitamin C induces a state of subfertility, reduces overall fecundity, and adversely impacts both pregnancy outcomes and growth in the offspring. The potential biological mechanisms that underlie these findings and the implications for future dietary recommendations in humans are discussed.

## 2. Materials and Methods

### 2.1. Ethics

This study was prospectively approved by the Animal Ethics Committee at the University of Otago, Wellington (AEC: 20–85). All procedures were performed in accordance with the Health Research Council of New Zealand code of practice for the care and use of animals for scientific purposes and results are reported according to the ARRIVE (animals in research: reporting in vivo experiments) guidelines [34].

### 2.2. Animal Model

Animals were sourced from the outbred colony of Dunkin Hartley guinea pigs maintained in the Biomedical Research Unit at the University of Otago Wellington. Females were group housed in floor pens and males were individually housed in cages under a 12 h day/night light cycle within a temperature (20–23 °C) and humidity (50–70%) controlled facility.

At 8 weeks of age, 73 females and 30 males were randomised into two weight-stratified dietary groups receiving optimal (900 mg/kg, males (*n* = 13), females (*n* = 35)) or low (100 mg/kg, males (*n* = 17), females (*n* = 38)) concentrations of vitamin C via purified guinea pig pellets (Specialty Feeds, Glen Forrest, Australia). The pellets were identical in all aspects except vitamin C content; a comprehensive list of diet ingredients can be found in the Supplementary Materials. A diet containing 100 mg of vitamin C/kg of feed has previously been shown to induce a non-scorbutic vitamin C deficiency in guinea pigs of all ages [35,36,37,38], including during pregnancy [23,39,40]. As expected, no animals developed signs of scurvy during the study [41]. Animals had ad libitum access to feed and filtered drinking water throughout the study. The diets were additionally supplemented with dried hay (~20 g per animal daily) that was devoid of vitamin C following autoclaving at 120 °C. Animals were monitored twice daily for general welfare (active and alert, not in isolation, decent food, and water intake) and weighed weekly by trained and familiar personnel.

Routine plasma sampling via ear vein blood collection was performed to confirm the response to the feeding regimens. Samples were collected from dams and sires at mating and from dams upon delivery of pups. To limit variations in plasma vitamin C concentrations due to recent food intake, animals were fasted for 2 h prior to all blood collections.

### 2.3. Breeding

Breeding began following a diet-acclimation period that lasted for at least 3 weeks. Previous studies in guinea pigs have shown that 3 weeks provides sufficient time for vitamin C concentrations to become stably established in plasma and tissue after altering dietary intake [23,38]. To ensure timed matings of virgin guinea pigs, we tracked the oestrous cycles of female animals by making daily observations of the vaginal membrane for at least 2 weeks prior to mating. The length of a guinea pigs’ oestrous cycle is 16 days on average [42]. During the latent phase of the cycle, the vaginal membrane is pale in colour and appears visibly closed. In the days leading up to ovulation, a noticeable colour change occurs with the vaginal membrane becoming progressively darker before spontaneously perforating at the time of ovulation. Upon noting the colour change and first signs of perforation, females were housed with a non-lineage and diet-matched male for 48 h. The date of conception/gestational age (GA) 0 was deemed as the middle 24 h of this mating period. Pregnancies were confirmed and litter size (*n* pups) was determined via abdominal ultrasound, detectable within the first trimester on ~gestational day (GD) 21.

### 2.4. Delivery and Postnatal Care of Offspring

Dams delivered spontaneously on ~GD 69 and pups were randomised into three weight-stratified groups: euthanised within 24 h of birth (neonate), euthanised at 28 days of age (juvenile), or euthanised at 4 months of age (adolescent). Offspring were further classified according to the dietary vitamin C status of their parents as either ‘optimal’ or ‘low’. The experimental groups were, therefore, neonate optimal males (*n* = 23), neonate low males (*n* = 19), neonate optimal females (*n* = 22), neonate low females (*n* = 18), juvenile optimal males (*n* = 13), juvenile low males (*n* = 10), juvenile optimal females (*n* = 11), juvenile low females (*n* = 10), adolescent optimal males (*n* = 8), adolescent low males (*n* = 7), adolescent optimal females (*n* = 9), and adolescent low females (*n* = 7). To prevent litter bias, no more than 2 pups/sex/litter were allocated to the same group.

After delivery, all mothers and pups were maintained on the optimal (900 mg of vitamin C/kg feed) diet. This approach ensured that any observed effects in the offspring were solely attributable to prenatal depletion of vitamin C. Mothers and pups were housed together in a cage during the neonatal period (first 7 days of life) for close monitoring and then group housed in a nursery pen with other mothers and pups until weaning. Saliva samples, obtained by allowing animals to chew on the tip of a cotton bud, and fasted plasma samples, obtained via ear vein blood sampling, were collected from dams and pups within 24 h of delivery. Pups were weighed daily for the first 7 days and subsequent plasma samples were collected from pups on day 7.

While the focus of this study is the impact of variation in foetal vitamin C exposure on the neonate, relevant data were pooled from all three offspring groups where possible, e.g., birth weights and anthropometric body measurements. For offspring growth trajectories during the neonatal period, data are pooled from the juvenile and adolescent groups.

### 2.5. Euthanasia and Tissue Collection

Adult animals were fasted for at least 4 h and pups were fasted for at least 2 h prior to euthanasia. Euthanasia was then performed by exsanguination under isoflurane (AttaneTM, Bayer Australia Ltd., Bentley, WA, Australia). An intracardiac blood sample was collected and organs were dissected, weighed, and snap-frozen in liquid nitrogen. For adults (*n* = 21), tissue sampling of major organs, including the testes (see Supplementary Figure S1 and Table S3 for a list of all sampled organs), was performed on virgin animals following the diet-acclimation period that lasted for at least 3 weeks. Ovaries (*n* = 12) were sampled post-partum, within 24 h of delivery.

### 2.6. High-Performance Liquid Chromatography (HPLC)

Blood samples (obtained via the ear vein or intracardially during exsanguination) were stored on ice until centrifugation and plasma was subsequently frozen at −80 °C. Tissue samples were snap-frozen in liquid nitrogen at the time of collection and subsequently stored at −80 °C. Frozen plasma and tissue samples were analysed for vitamin C content by high performance liquid chromatography with electrochemical detection (HPLC-ECD) using a previously published method [43,44,45].

### 2.7. Cortisol Enzyme Linked Immunosorbent Assay (ELISA)

Saliva samples were obtained from dams and pups on the day of delivery/birth and were stored at 4 °C. The Salimetrics salivary cortisol assay (Salimetrics Inc., State College, PA, USA) was used to measure cortisol concentrations in guinea pig saliva samples as per the manufacturer’s instructions. The sensitivity of the assay was 0.012–3.0 μg/dL and the inter- and intra-assay coefficients of variance were 8.6% and 5.1%, respectively.

### 2.8. Statistical Analyses

All data were analysed using GraphPad Prism software (version 9.2.0). All tests were two-tailed with statistical significance considered as *p* < 0.05. Means and standard deviations (SD) were used in the analysis of continuous variables and counts with proportions were used in the analysis of categorical variables. Normality was assessed using the Kolmogorov–Smirnov normality test and equality of variance was assessed using the F test or Brown–Forsythe test. For adult breeding animals, group differences were assessed within sex using unpaired *t*-tests or Fisher’s exact tests to compare proportions. For offspring, group differences were assessed using two-way ANOVA with diet and sex as factors. A multi-factorial design was chosen as it can account for a larger proportion of variability using smaller sample sizes (an ethical priority when using animal models) than single-factor analysis allows [46]. If the two-way ANOVA indicated a significant effect of diet (*p* < 0.05), subsequent post-hoc tests with Šidák’s correction for multiple comparisons were performed. *p*-values for multiple comparisons are presented in the text while overall model *p*-values are provided in the supplementary materials. For data that were not normally distributed, the Mann–Whitney or Kruskal–Wallis tests were used and for data with unequal variance, Welch’s correction was applied.

## 3. Results

### 3.1. Adult Physical Characteristics

Mean weights at the study enrolment, mean weights, and ages at mating and mean gonad weights for adult guinea pigs maintained on optimal and low vitamin C diets are presented in Table 1 (see also Supplementary Table S1). No significant differences were observed between groups.

### 3.2. Vitamin C Concentrations in Adults

#### 3.2.1. Plasma

**Figure 1:**
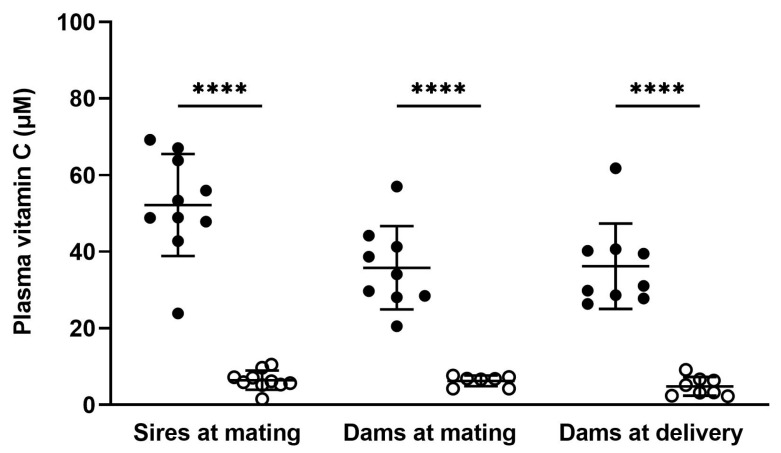
Plasma vitamin C concentrations in parent animals on the day of mating and day of delivery. At mating, optimal sires and dams (full circles, *n* = 10 male, *n* = 9 female) and low sires and dams (open circles, *n* = 10 male, *n* = 7 female). At delivery, optimal dams (full circles, *n* = 9) and low dams (open circles, *n* = 8). Data are presented as individual points with group means ± SD. An asterisk indicates significance between bars linked with black lines; **** = *p* < 0.0001. Data were analysed using unpaired *t*-tests with Welch’s correction for heterogeneity of variance applied.

Plasma vitamin C concentrations were assessed in a random selection of sires and dams on the day of mating and in dams on the day of delivery (Figure 1 and Supplementary Table S2). Optimal sires had higher plasma vitamin C concentrations on the day of mating compared to low sires (*p* < 0.0001). Optimal dams had higher plasma vitamin C concentrations on the day of mating and on the day of delivery compared to low dams (*p* < 0.0001 for both timepoints). Optimal sires also had higher plasma vitamin C concentrations on the day of mating when compared to optimal dams (*p* = 0.0096). This difference between males and females with optimal vitamin C intake may be due to the increased turnover of vitamin C that occurs in females around the time of ovulation [2,3,4,5,6,7].

#### 3.2.2. Tissue

**Figure 2:**
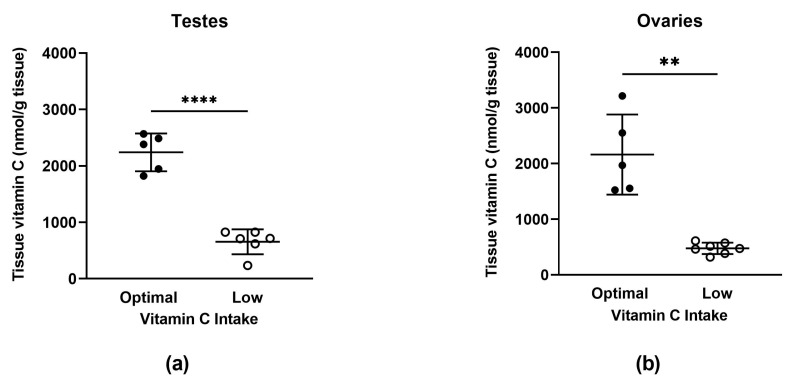
Vitamin C concentrations in testes (**a**) and ovaries (**b**). Optimal (full circles, *n* = 5 testes, *n* = 5 ovaries) and low (open circles, *n* = 6 testes, *n* = 7 ovaries). Data are presented as individual points with group means ± SD. An asterisk indicates significance between bars linked with black lines; ** = *p* < 0.01 and **** = *p* < 0.0001. Data were analysed using unpaired *t*-tests (with Welch’s correction for heterogeneity of variance applied to ovaries).

Vitamin C concentrations were measured in the testes and ovaries (Figure 2a,b) and other major organs (Supplementary Figure S1). Animals on the low vitamin C diet had significantly decreased vitamin C concentrations in the testes (*p* < 0.0001) and ovaries (*p* = 0.006) compared to animals on the optimal vitamin C diet. This decrease in tissue vitamin C concentration was also observed consistently across the other major organs (see Supplementary Figure S1).

### 3.3. Fertility Parameters

To measure the effect of vitamin C intake on fertility, reproductive parameters were measured throughout the study. These parameters included oestrous cycle length, the number of unsuccessful matings, failure to establish pregnancy, and failure to sire progeny (Table 2 and Supplementary Table S4). Females were removed from the study due to a failure to establish pregnancy at six months of age which allowed for between four and five mating attempts with a proven male. Males were removed from the study due to a failure to sire progeny after a minimum of six unsuccessful matings between at least two different females. The overall reproductive success rate was calculated by dividing the number of guinea pigs that became pregnant (females) or were proven able to sire progeny (males), by the total number of guinea pigs eligible to become pregnant or sire progeny. The proportion of matings that were unsuccessful, i.e., did not result in a pregnancy, was higher in the low vitamin C group compared to the optimal group (51.7% vs. 34.9%, *p* = 0.0228). Other parameters were unaffected.

### 3.4. Pregnancy Weight Gain

**Figure 3:**
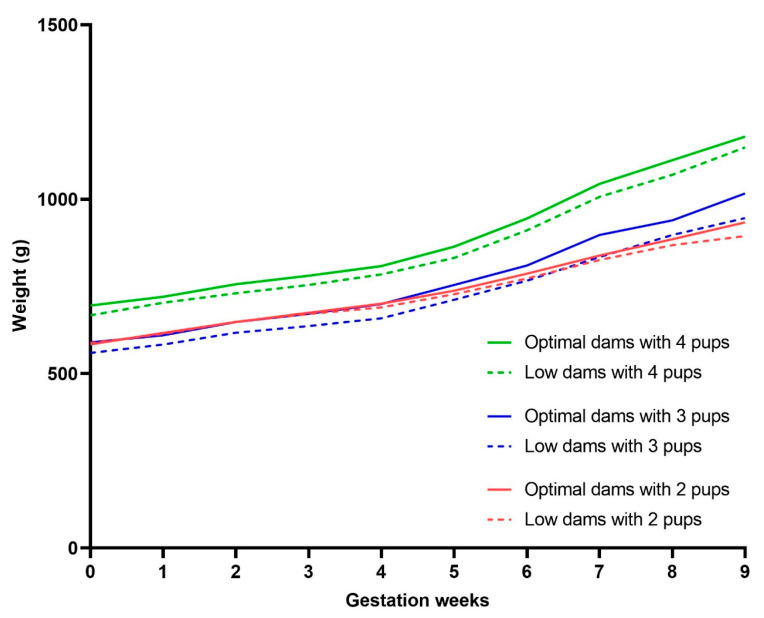
Pregnancy weight gain in litters of equal size. The graph represents the average weight gain during pregnancy (day of mating to week of delivery). Full lines = optimal dams and dashed lines = low dams. Dams with 4 pups (green lines, *n* = 10 optimal, *n* = 6 low), dams with 3 pups (blue lines, *n* = 11 optimal, *n* = 10 low), and dams with 2 pups (red lines, *n* = 6 optimal, *n* = 12 low). Data were analysed using repeated measures mixed-effects two-way ANOVA.

To observe any differences in weight gain during pregnancy, dams were weighed weekly. The growth trajectory was similar between groups during the first five weeks of pregnancy (Supplementary Figure S2 and Supplementary Table S5). From the sixth week, optimal and low dams began to diverge, with optimal dams showing significantly greater weight gain compared to low dams (week 6; *p* = 0.0305, week 7; *p* = 0.0099, week 8; *p* = 0.0145, week 9; *p* = 0.0042). However, to control for differences in litter size (*n* pups), further comparisons were made within dams carrying the same number of pups (Figure 3). Only dams carrying litters of 2–4 pups were included as this is typical for guinea pigs and sample sizes were comparable between groups. The difference in pregnancy-associated weight gain between low and optimal dams was no longer apparent once corrected for litter size.

### 3.5. Pregnancy Outcomes

To measure the effect of vitamin C intake on general pregnancy and perinatal outcomes, rates of pregnancy loss/miscarriage, foetal reabsorption, preterm delivery, and stillbirth, as well as litter size, gestational age (GA) of pups at delivery, litter birth weights, and pup sex ratios were assessed (Table 3 and Supplementary Table S6).

Delivery of pups earlier than GA 62 (term delivery ~GA 69) normally results in stillbirth or early neonatal mortality: pups liveborn at this gestational age require neonatal resuscitation and extensive intervention to survive. For the purposes of this study, spontaneous delivery before GA 62 was classified as a miscarriage. Litters born between GA 62 and GA 66 can be viable without intervention but are nevertheless classified as premature. Foetal reabsorption was evidenced by a greater number of pups detected at ultrasound compared to pups delivered, paired with significant maternal weight loss or weight stagnation during pregnancy and/or delivery of partially reabsorbed pups.

Due to the potential cannibalism of underdeveloped pups that occurs in guinea pigs, miscarriages and premature deliveries were excluded from the analysis of litter size (*n* pups). Since litter size can influence both the duration of pregnancy and pup birth weights, GA of pups at delivery and average litter birth weight only include term litters of between two and four pups, which is typical for guinea pigs.†

The rate of foetal reabsorption was higher in low vitamin C pregnancies compared to optimal (25% vs. 5.9%, *p* = 0.0463). The cumulative adverse pregnancy outcome rate which included miscarriages, foetal reabsorption, preterm deliveries, and stillbirth (# of pregnancies) was also higher in the low vitamin C group (61.1% vs. 32.4%, *p* = 0.0188). Pregnancies characterised by low vitamin C status also produced significantly fewer offspring in terms of litter size (*n* pups) compared to optimal (*p* = 0.0061). Other parameters were not affected by low vitamin C status.

### 3.6. Offspring Physical Characteristics

On the day of delivery/birth (day zero), body weights and measurements were recorded and are displayed below in Table 4 (see also Supplementary Table S7). Maternal vitamin C intake had no significant effect on birth weights for offspring of either sex. For body measurements, there were no significant differences in the crown-rump length (CRL) or hock-toe length (HT). However, maternal vitamin C depletion affected hind limb length (HL) in males. Hind limb length was significantly shorter in males born to low vitamin C mothers compared to optimal (*p* = 0.003). The ponderal index (PI), an estimate of adiposity in relation to length, was calculated using the following formula: weight (kg)/length (m)^3^. The sum of a guinea pig’s CRL and HL was used as an approximation of the animal’s height. Average PI-derived adiposity at birth was greater in pups born to low vitamin C mothers (males; *p* = 0.0418 and females; *p* = 0.0077). At the time of neonate tissue collection (within 24 h of delivery), organ weights were recorded and are displayed below in Table 5 (see also Supplementary Table S7). Maternal vitamin C intake had no significant effect on relative organ weights for offspring of either sex. There was a significant effect of sex on relative visceral fat weights observed in optimal offspring (*p* = 0.0010). No other significant differences were identified.

**Figure 4:**
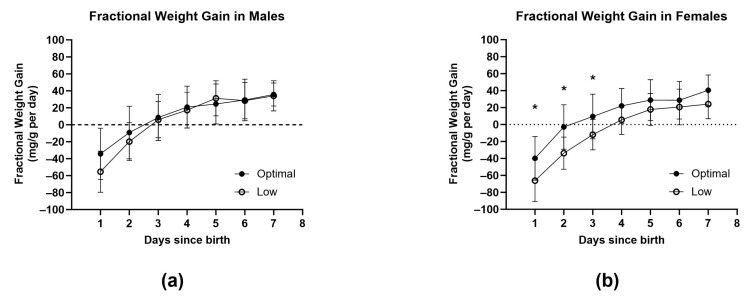
Fractional weight gain in male (**a**) and female (**b**) offspring during the neonatal period. Optimal offspring (full circles, *n =* 21 males and *n* = 20 females) and low offspring (open circles, *n* = 17 males and *n* = 17 females). Data are presented as group means ± SD with * indicating significance at *p* < 0.05. Data were analysed using repeated measures of mixed-effects two-way ANOVA.

All pups allocated to the juvenile or adolescent groups were extensively monitored for the first seven days. A key indicator of health and wellbeing during the neonatal period is daily fractional weight gain (Figure 4a,b and Supplementary Table S8). Low maternal vitamin C intake significantly impaired fractional weight gain for females compared to their optimal counterparts during the first three days of life (postnatal day one; *p* = 0.0339, postnatal day two; *p* = 0.0046, and postnatal day three; *p* = 0.0490).

### 3.7. Vitamin C Concentrations in Offspring

#### Plasma

**Figure 5:**
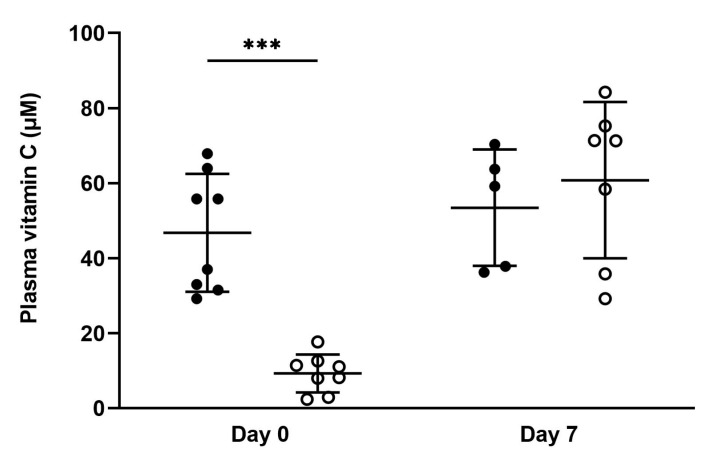
Plasma vitamin C concentrations in neonate offspring (pooled males and females) on day 0 and day 7. At day 0, optimal offspring (full circles, *n* = 8) and low offspring (open circles, *n* = 8). At day 7, optimal offspring (full circles, *n* = 5) and low offspring (open circles, *n* = 7). All data are presented as group means ± SD. An asterisk indicates significance between bars linked with black lines; *** = *p* < 0.001. Data were analysed using unpaired *t*-tests (with Welch’s correction for heterogeneity of variance applied to day 0).

Plasma vitamin C concentrations were assessed in pups (pooled males and females) on the day of delivery (day zero) and on day seven (Figure 5 and Supplementary Table S9). Pups born from low vitamin C pregnancies had decreased plasma vitamin C concentrations on day zero when compared to pups born from optimal vitamin C pregnancies (*p* = 0.0002). This effect had normalised by day seven on the vitamin C-replete diet, with no significant differences in plasma vitamin C concentrations observed between groups at this age.

### 3.8. Salivary Cortisol Concentrations

**Figure 6:**
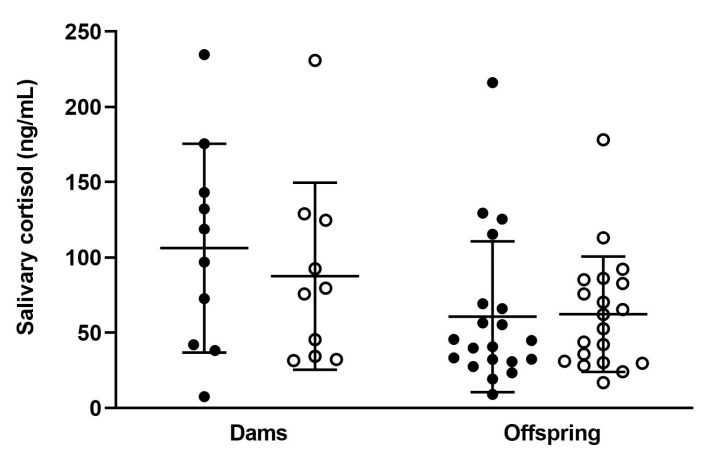
Postpartum salivary cortisol concentrations in dams and offspring. Optimal dams and offspring (full circles, *n* = 10 dams and *n* = 20 pups) and low dams and offspring (open circles, *n* = 10 dams and *n* = 20 pups). All data are presented as group means ± SD and were analysed using one-way ANOVA.

Salivary cortisol was assessed in dams and pups on the day of delivery/birth (day 0). There was no significant effect of maternal vitamin C intake on postpartum salivary cortisol concentrations in dams or offspring as shown in Figure 6 (see also Supplementary Table S10). Neonate concentrations were also in the expected range based on previous guinea pig data [47]. This implies that there were no overt birth complications causing elevated stress in pups during labour or delivery in either group.

## 4. Discussion

The data presented in this study represent the first comprehensive characterisation of the adverse fertility and pregnancy outcomes associated with low but not scorbutic levels of vitamin C prior to and during pregnancy in a species that shares the same dietary requirements for vitamin C as humans. Given the dramatic impact that modifiable lifestyle factors such as diet can have on natural fertility, the effectiveness of infertility treatments, and pregnancy and neonatal outcomes, it is essential that we select the right experimental paradigm to accurately study the effects of dietary variations on reproductive health. Indeed, the strengths of our study are grounded in the clinical relevance of using guinea pigs as a model for reproductive and developmental research. Guinea pigs not only serve as an excellent model for human pregnancy and perinatal development, as previously reviewed [48], but also represent a natural model of diet-induced vitamin C deficiency [1]. This enhances the translatability of our findings to potential human health implications. Nevertheless, certain limitations must also be recognised. While the guinea pig provides a valuable and necessary framework, it is not a perfect proxy for humans. Further research is necessary to directly apply these findings to other animal species or even to diverse human populations with distinct genetic and environmental factors.

In accordance with previous work [23,35,36,37,38,39,40], we show that a diet containing 100 mg of vitamin C per kg of feed successfully induces a non-scorbutic vitamin C deficiency in guinea pigs of both sexes and of various adult ages, including during pregnancy. Moreover, this depletion was systemic, with animals maintained on the low vitamin C diet exhibiting markedly decreased vitamin C concentrations in plasma, reproductive tissue, and other organs (Figure 1 and Figure 2 and Supplementary Figure S1). Our rationale for having both parent animals on matched diets was based on the assumption that, in most human couples, dietary patterns of mothers and fathers tend to be relatively similar. There were also practical considerations that came into play. Males were used for breeding with multiple females throughout the study; constantly switching between the optimal and low vitamin C diets would not only have made it difficult to accurately assess their true vitamin C status but also raised ethical concerns regarding the animals’ welfare.

Low vitamin C intake had a significant impact on the number of unsuccessful matings in our study. Over half of the total matings in the low vitamin C group (51.7%) did not result in a pregnancy compared to 34.9% in the optimal group. Pregnancies may have been established in some cases but embryonic loss occurred before ultrasounds took place on ~GD 21 and the pregnancy went undetected. These results imply subfertility or low fecundability in pairings characterised by low vitamin C status. As we did not monitor actual copulation events, it is possible that vitamin C-depleted guinea pigs failed to manifest sexual instincts or had reduced sexual activity. It is also possible that low vitamin C concentrations within reproductive tissues impaired reproductive ability through perturbations in semen function, follicle development, ovulation, fertilisation, or endometrial function and reduced the likelihood of mating resulting in pregnancy.

Previous research has demonstrated rapid degeneration of the entire male reproductive system [49] and ovarian atrophy [50] in scorbutic guinea pigs. In humans, several studies have reported improvements in various reproductive parameters following vitamin C supplementation. For instance, men diagnosed with oligospermia experienced an increased total sperm count, sperm motility, and normal sperm percentage after vitamin C supplementation [20]. Similar improvements have been observed in the male partners of couples experiencing recurrent pregnancy loss [51] and in obese men [52]. Vitamin C also regulates spermatogenesis via epigenetic mechanisms. The ten–eleven translocation (TET) enzymes (TET 1,2,3) are a family of DNA demethylases that require vitamin C for optimal catalytic activity [53,54,55]. Men diagnosed with oligospermia and/or asthenospermia showed significantly reduced TET enzyme expression in their sperm [56]. Interestingly, TET expression also showed a positive correlation with fertilisation rates among couples undergoing intracytoplasmic sperm injection (ICSI) infertility treatment [56].

Among women of the child-bearing age, a leading cause of subfertility is polycystic ovarian syndrome (PCOS), a common endocrine disorder linked to disordered follicle development and diminished ovulation [57,58]. Whether vitamin C supplementation can improve ovarian pathophysiology associated with PCOS is an emerging area of research [8,10]. However, vitamin C has already been shown to enhance the ovulation-inducing effects of clomiphene treatment in anovulatory women [59]. A luteal phase defect (LPD) is another endocrine disorder affecting women that is associated with severe subfertility and spontaneous miscarriage. Characterised by low progesterone levels, LPD inhibits the normal endometrial development necessary for embryonic implantation and pregnancy maintenance [11,12]. A study in women diagnosed with LPD indicated that vitamin C supplementation can increase serum progesterone concentrations to healthy control levels and improve the clinical pregnancy rate [12]. It is inevitable that most human fertility research focuses on specific reproductive issues as individuals who do not experience fertility problems generally do not seek treatment. Our findings highlight that adequate vitamin C intake during the preconception period holds the potential for improving fertility outcomes for all prospective mothers and fathers.

Another key finding from our study is that low vitamin C intake had a significant impact on the incidence of early foetal loss. Foetal reabsorption was evident in 25% of low vitamin C pregnancies compared to 5.9% of optimal pregnancies. Previous studies in guinea pigs have also reported an increase in the number of reabsorbed foetuses in vitamin C deficient mothers at mid-gestation [14,60]. In humans, early pregnancy loss or miscarriage occurring in the first trimester affects 10–20% of clinically recognised pregnancies [61]. However, this is likely an underestimation of the true incidence as many miscarriages occur within the first month before a woman knows she is pregnant. In many cases, early pregnancy loss is believed to be due to embryonic or foetal abnormalities that cause developmental arrest [62]. However, given the inherent complexity of researching early pregnancy loss, many unresolved questions remain, especially because for many women, medical investigation into early pregnancy loss is not initiated until several cycles of conception and loss have been completed. Developmental arrest poses a serious challenge for assisted reproductive technologies (ART) such as in vitro fertilisation (IVF), affecting up to 50% of in vitro embryos [62]. Identifying and understanding the molecular mechanisms that drive developmental arrest has important implications for improving ART outcomes.

The TET enzymes remain highly expressed during early mammalian development where they act as pivotal regulators of gene expression by catalysing the oxidation of methylcytosine to 5-hydroxymethylcytosine and subsequent derivatives [53]. Studies in mice have revealed that knockout of Tet1 and Tet3, alone or in combination, significantly reduces the likelihood of a normal trajectory of foetal development by accelerating rates of embryonic death and morphological abnormalities that lead to developmental arrest [63,64]. One possible mechanism at play in our model of vitamin C depletion is reduced TET catalytic activity resulting in the perturbation of key gene expression pathways during early development. Compared to the complete knockout of individual TET enzymes, low maternal vitamin C intake might cause a more subtle dampening of TET activity and affect all three paralogs simultaneously.

Standard cell culture media such as RPMI does not typically include vitamin C and vitamin C is quickly lost from solution after being added [45]. Supplementing vitamin C to culture media has been shown to enhance TET activity in IVF mouse embryos. Moreover, implantation success and post-implantation survival rates were increased following embryo transfer when vitamin C had been added to culture media [65]. Enhanced TET activity with vitamin C supplementation has also been widely reported in a plethora of different cell lines [45,54]. Commercially available culture media for human IVF does contain vitamin C but, due to the competitive nature of the field and a handful of companies monopolising the market, concentrations are not disclosed [66]. Whether vitamin C could be used as an inexpensive and readily available adjunct to infertility treatments to improve preimplantation embryo quality and clinical pregnancy rates is an exciting prospect.

We found that dams maintained on the low vitamin C diet gave birth to significantly fewer offspring (n born per litter), which may be due in part to the increased rate of foetal reabsorption. This shows that while animals with low vitamin C intake can be fertile, they have significantly reduced fecundity. In a study by DiTroia et al. that employed a Gulo knockout mouse model of vitamin C deficiency, the authors found that maternal vitamin C deficiency during pregnancy significantly impaired reproductive performance in the F1 generation [67]. Female offspring born to vitamin C-deficient mothers had an increased number of unsuccessful matings and reabsorbed embryos during their own pregnancies. This is extremely interesting given that these findings mirror what we have observed in our study of reproduction in the F0 generation.

A key finding from the DiTroia study was the similarity between the transcriptomes of Tet1 knockout mice and germ cells in embryos from vitamin C deficient mothers [67]. This observation implies that a deficiency in vitamin C during pregnancy phenocopies the loss of Tet1 in the Gulo mouse context. Moreover, an analysis of differentially methylated CpG sites in the genomes of vitamin C deficient germ cells within Gulo mouse embryos highlighted an enrichment of genomic regions associated with fertility related pathways (such as ovarian folliculogenesis, ovary morphology, and ovarian follicle number). A possible mechanism implied by this work is the aberrant regulation of methylation at genomic regions associated with fertility-related functions in the F1 generation following vitamin C deficiency [67]. Similar mechanisms may be at play in our study, albeit in the F0 generation. This potentially highlights an intergenerational mechanism for adapting fecundity in response to adverse environmental conditions such as low vitamin C availability.

Body measurements and organ weights taken at birth can provide valuable information on foetal growth and development. Males born to low vitamin C mothers had shorter hind limbs at birth, suggesting stunted or delayed limb growth in utero. Surprisingly, the average adiposity at birth was greater in pups born to low vitamin C mothers. These pups also had slightly greater birth weights, although this difference was not statistically significant. This may be attributed to the fact that low vitamin C dams gave birth to smaller litters (*n* pups), resulting in less growth restriction for the individual pups in utero and leading to larger sizes at birth. In females born to low vitamin C mothers, daily fractional weight gain was reduced during the first three days of life (Figure 4). This decreased weight gain may reflect a reduction in the level of activity that prevented active suckling from their mother. It could also indicate impaired bioenergetic capacity, resulting in reduced nutrient absorption or cellular respiration, leading to lower nutritional value from the same caloric intake.

Pups born to low vitamin C mothers had significantly decreased plasma vitamin C concentrations on the day of delivery/birth (Figure 5). Following delivery, all mothers and pups were provided with the vitamin C-replete diet and by postnatal day seven, plasma vitamin C concentrations in the depleted new born offspring had increased to healthy control levels (Figure 5). Since breastmilk is the major source of nutrition for guinea pig pups during early postnatal life, this observation demonstrates that the lactation period is an effective time to implement potential therapies, e.g., vitamin C supplementation, to try and mitigate any ongoing adverse effects caused by foetal vitamin C depletion. Indeed, Kawahori et al. have shown in a genetically engineered mouse model of vitamin C deficiency, that the administration of high dose vitamin C to deficient dams during the lactation period appeared to restore Tet function in offspring by increasing global liver 5-hydroxymethylcytosine (a marker of increased Tet activity) [68]. However, it is important to note that the new born mouse is born in a developmentally immature state compared to either humans or guinea pigs, so the extent to which lactational effects in this species can be translated to the human setting requires further evaluation.

The recommended dietary intake (RI) for human infants between birth and six months of age is 40 mg of vitamin C/day [69]. This is easily achieved through responsive breastfeeding if the mother is consuming an adequate amount of the vitamin [70]. The highest vitamin C concentrations are found in colostrum [70,71], indicating that the neonates’ immediate requirement for vitamin C is substantial [72,73]. Infant formulas also contain vitamin C [72,73]. However, storage can lead to vitamin C degradation, decreasing the nutritional content of bottled formula or breastmilk [73]. In well-nourished mothers, mature breastmilk contains 50–90 mg of vitamin C/L and this concentration remains relatively stable until around 12 months postpartum [71,74]. In poorly nourished mothers with low vitamin C intake, breastmilk concentrations can be as low as 20–30 mg/L [75,76], putting the exclusively breastfed infant at risk of deficiency.

These findings, in conjunction with our own observations, emphasise the critical importance of adequate maternal vitamin C intake not only during pregnancy but also throughout the postpartum period, especially during the perinatal and neonatal stages. This is particularly crucial for breastfeeding mothers with a heightened risk of vitamin C deficiency, such as those who smoke. Smoking is known to have a negative effect on plasma and tissue vitamin C concentrations, which is reflected in lower breast milk vitamin C concentrations among smoking mothers [70,77]. In Australasia, the recommended dietary intakes for pregnant and breastfeeding women are relatively low, at 60 and 85 mg of vitamin C/day, respectively. In other countries such as China and the United States, the respective amounts are considerably higher at 85 and 120 mg of vitamin C/day [69]. A review of the current reference values for daily vitamin C intake in New Zealand is long overdue [78,79,80]. Focus should be given to pregnant and breastfeeding women with demographic and lifestyle factors such as smoking considered. Optimising nutrient availability during the perinatal and neonatal periods will ensure future generations are set on the best possible trajectory to achieve long-term health.

## 5. Conclusions

The findings of our study indicate that in a species reliant on dietary vitamin C, low vitamin C status reduces fecundability, may increase the time to pregnancy, and results in altered offspring characteristics. Given the similarities between guinea pigs and humans in terms of reproductive physiology, e.g., guinea pigs are polyoestrous and nonseasonal breeders, this may have implications for human couples who are trying to conceive. Furthermore, our findings suggest that low vitamin C status during periconception and pregnancy might increase the risk of embryonic or foetal loss, resulting in an overall reduction in fecundity, and lead to compromised foetal and neonatal health. Whilst severe dietary deficiencies would be expected to compromise the outcome of any pregnancy, it is alarming that non-scorbutic intakes of vitamin C which have no apparent effect on the outward physical health of adult animals and can have such a dramatic impact on fertility and pregnancy and neonatal outcomes. Our results highlight the importance of adequate vitamin C intake well above the concentrations required to prevent the overt symptoms of deficiency such as scurvy. Key timeframes during which adequate vitamin C intake is important include periconception, pregnancy, and lactation. Future studies should investigate the long-term outcomes for offspring as well as outcomes for offspring that are also depleted postnatally as this would likely be the case for exclusively breastfed infants born to mothers with low vitamin C status. Additionally, the impact of dietary mismatch between males and females prior to mating and the impact of changing vitamin C availability during pregnancy all offer unique translational insights into the impact of vitamin C as a cheap and easily modifiable dietary strategy to improve pregnancy outcomes in women.

## Figures and Tables

**Table 1:** Adult Physical Characteristics.

Characteristics	Optimal Vitamin C	(*n*)	Low Vitamin C	(*n*)
Male weight (g) at enrolment	463.3 ± 86.3	13	489.5 ± 57.9	17
Sire weight (g) at mating	744.6 ± 132.4	34	734.9 ± 104.4	36
Sire age (weeks) at mating	16.5 ± 3.1	34	17.8 ± 3.6	36
Female weight (g) at enrolment	451.9 ± 49.0	35	432.7 ± 48.1	38
Dam weight (g) at mating	632.7 ± 111.8	34	588.5 ± 85.9	36
Dam age (weeks) at mating	14.9 ± 4.0	34	16.1 ± 5.2	36
Testis-to-body weight	0.228 ± 0.030	5	0.217 ± 0.029	6
Ovary-to-body weight	0.011 ± 0.003	5	0.009 ± 0.002	7

Weights for testes and ovaries were averaged for each animal and are expressed as a percentage of body weight on the day of euthanasia. All data are presented as group means ± SD. Data were analysed using unpaired *t*-tests (or the Mann–Whitney test for dam age at mating).

**Table 2:** Fertility Parameters.

Characteristics	Optimal Vitamin C	(*n*)	Low Vitamin C	(*n*)
Oestrous cycle length (days)	15.9 ± 0.7	35	15.8 ± 0.7	38
Unsuccessful matings (*n* = total matings)	30 (34.9%)	86	62 (51.7%) *	120
Failure to establish pregnancy (*n* = total females)	1 (2.9%)	35	2 (5.3%)	38
Failure to sire progeny (*n* = total males)	1 (7.7%)	13	4 (23.5%)	17
Reproductive success rate (*n* = total animals)	95.8%	48	89.1%	55

Oestrous cycle length is presented as group means ± SD and was analysed using the Mann–Whitney test. Unsuccessful matings, failure to establish pregnancy, and failure to sire progeny are presented as absolute counts with the proportion of the total shown in brackets. The overall reproductive success rate includes the total animals that were enrolled in the study. Fisher’s exact test was used to compare proportions. * indicates significance at *p* < 0.05.

**Table 3:** Pregnancy Outcomes.

Outcomes	Optimal Vitamin C	(*n*)	Low Vitamin C	(*n*)
Miscarriage (delivery before GA62) (*n* = total pregnancies)	4 (11.8%)	34	2 (5.6%)	36
Foetal reabsorption (*n* = total pregnancies)	2 (5.9%)	34	9 (25%) *	36
Preterm delivery (born GA62–66) (*n* = litters born GA62+)	0	30	2 (5.9%)	34
Stillbirth # of pregnancies (*n* = litters born GA62+)	5 (16.7%)	30	9 (26.5%)	34
Cumulative adverse outcome (*n* = total pregnancies)	11 (32.4%)	34	22 (61.1%) *	36
Stillbirth # of pups (*n* = total pups)	6 (5.9%)	102	11 (13.1%)	85
Litter size (*n* pups) (*n* = term litters (born GA66+))	3.4 ± 1.0 (2–6)	30	2.6 ± 0.9 (1–4) *	32
GA of pups at delivery (*n* = term litters of 2–4 pups) †	68.6 ± 1.0	27	68.9 ± 1.2	27
Litter birth weight (g) (*n* = term litters of 2–4 pups) †	95.3 ± 10.4	27	97.2 ± 10.0	27
Pup sexes (*n* = total liveborn pups)	49♂ 47♀	96	37♂ 37♀	74

Rates of miscarriage, foetal reabsorption, preterm delivery, stillbirth (# of pregnancies and # of pups), and cumulative adverse outcome are presented as absolute counts with the proportion of the total shown in brackets. Litter size is presented as group means ± SD with smallest to largest litter sizes shown in brackets. The GA of pups at delivery and litter birth weight are presented as group means ± SD. Data were analysed using unpaired *t*-tests (or the Mann–Whitney test for litter size and GA of pups at delivery). Fisher’s exact test was used to compare proportions. * indicates significance at *p* < 0.05. † One optimal litter of 6, two optimal litters of 5, three low litters of 1, and two low premature litters were excluded from the analysis of GA of pups at delivery and litter birth weight.

**Table 4:** Body Weights and Measurements on Day Zero.

Characteristics	Males		Females	
	Optimal (*n* = 44)	Low (*n* = 36)	Optimal (*n* = 42)	Low (*n* = 35)
Body weight (g)	93.3 ± 14.8	96.3 ± 13.2	91.4 ± 12.9	93.5 ± 14.8
Crown-rump (mm)	128.8 ± 8.8	129.0 ± 9.0	127.7 ± 8.7	126.0 ± 8.7
Hind limb (mm)	38.5 ± 5.0	35.1 ± 3.7 **	36.7 ± 4.3	34.7 ± 3.6
Hock-toe (mm)	38.2 ± 5.1	36.8 ± 3.1	37.3 ± 4.6	35.0 ± 3.6
Ponderal Index (PI)	20.2 ± 3.4	22.3 ± 2.9 *	20.6 ± 3.3	23.2 ± 2.7 **

All data are presented as group means ± SD and were analysed as a 2 × 2 factorial design with sex*diet*interaction included. An asterisk indicates significance within sex; * = *p* < 0.05 and ** = *p* < 0.01. These data represent all new born pups irrespective of later group allocation.

**Table 5:** Organ Weights on Day Zero.

Characteristics	Males		Females	
	Optimal (*n* = 23)	Low (*n* = 19)	Optimal (*n* = 22)	Low (*n* = 18)
Brain-to-body wgt	2.67 ± 0.43	2.58 ± 0.29	2.71 ± 0.46	2.52 ± 0.30
Liver-to-body wgt	3.94 ± 0.74	3.79 ± 0.36	4.26 ± 0.56	3.88 ± 0.52
Brain-to-liver ratio	0.71 ± 0.19	0.68 ± 0.11	0.66 ± 0.16	0.67 ± 0.11
Heart-to-body wgt	0.41 ± 0.04	0.40 ± 0.05	0.43 ± 0.04	0.40 ± 0.03
Kidney-to-body wgt	0.43 ± 0.04	0.44 ± 0.04	0.43 ± 0.04	0.44 ± 0.04
Adrenal-to-body wgt	0.03 ± 0.02	0.02 ± 0.01	0.02 ± 0.01	0.05 ± 0.07
Testis-to-body wgt	0.07 ± 0.05	0.07 ± 0.09	N/A	N/A
Subcut. Fat-to-body wgt	1.50 ± 0.5	1.40 ± 0.3	1.34 ± 0.4	1.30 ± 0.3
Visc. Fat-to-body wgt	1.06 ± 0.3	1.04 ± 0.4	0.73 ± 0.3	0.81 ± 0.2

All data are presented as group means ± SD. All weights are in grams and are expressed as percentages of body weight. Paired organ weights were averaged for each animal. Data were analysed as a 2 × 2 factorial design with sex*diet*interaction included (or the Mann–Whitney test for testes). Wgt = weight, subcut. = subcutaneous, visc. = visceral. These data derive from pups randomised at birth to neonatal tissue collection. N/A means not applicable.

## Data Availability

The original contributions presented in the study are included in the article/Supplementary Materials, further inquiries can be directed to the corresponding author.

## Supplementary Materials

The following supporting information can be downloaded at: https://www.mdpi.com/article/10.3390/nu15194107/s1, Figure S1: Adult Tissue Vitamin C Concentrations; Figure S2: Pregnancy Weight Gain; Table S1: Adult Physical Characteristics; Table S2: Adult Plasma Vitamin C Concentrations; Table S3: Adult Tissue Vitamin C Concentrations; Table S4: Fertility Parameters; Table S5: Pregnancy Weight Gain; Table S6: Pregnancy Outcomes; Table S7: Relative Organ Weights; Table S8: Fractional Weight Gain; Table S9: Offspring Plasma Vitamin C Concentrations; Table S10: Salivary Cortisol Concentrations.

## References

[1] Drouin G., Godin J.-R., Page B. (2011). The genetics of vitamin C loss in vertebrates. Curr. Genom..

[2] Paeschke K.D. (1970). Ovulation preliminaries and ovulation. I. Generative function of the ovary and ascorbic acid metabolism during the ovarian cycle. Fortschr. Geburtshilfe Gynakol..

[3] Das P.C., Das K.P., Bagchi K., Dey C.D. (1993). Evaluation of tissue ascorbic acid status in different hormonal states of female rat. Life Sci..

[4] Ranjan R., Ranjan A., Dhaliwal G., Patra R. (2012). l-Ascorbic acid (vitamin C) supplementation to optimize health and reproduction in cattle. Vet. Q..

[5] Michos C., Kiortsis D.N., Evangelou A., Karkabounas S. (2006). Antioxidant protection during the menstrual cycle: The effects of estradiol on ascorbic-dehydroascorbic acid plasma levels and total antioxidant plasma status in eumenorrhoic women during the menstrual cycle. Acta Obstet. Gynecol. Scand..

[6] Loh H., Wilson C. (1971). Relationship of human ascorbic-acid metabolism to ovulation. Lancet.

[7] Pillay A.P. (1940). Vitamin C and Ovulation. Ind. Med. Gaz..

[8] Olaniyan O.T., Femi A., Iliya G., Ayobami D., Godam E., Olugbenga E., Bamidele O., Mali P.C. (2019). Vitamin C suppresses ovarian pathophysiology in experimental polycystic ovarian syndrome. Pathophysiology.

[9] Luck M.R., Jeyaseelan I., Scholes R.A. (1995). Ascorbic acid and fertility. Biol. Reprod..

[10] Iervolino M., Lepore E., Forte G., Laganà A., Buzzaccarini G., Unfer V. (2021). Natural Molecules in the Management of Polycystic Ovary Syndrome (PCOS): An Analytical Review. Nutrients.

[11] Mesen T.B., Young S.L. (2015). Progesterone and the luteal phase: A requisite to reproduction. Obstet. Gynecol. Clin. N. Am..

[12] Henmi H., Endo T., Kitajima Y., Manase K., Hata H., Kudo R. (2003). Effects of ascorbic acid supplementation on serum progesterone levels in patients with a luteal phase defect. Fertil. Steril..

[13] Mumford S.L., Browne R.W., Schliep K.C., Schmelzer J., Plowden T.C., Michels K.A., Sjaarda L.A., Zarek S.M., Perkins N.J., Messer L.C. (2016). Serum Antioxidants Are Associated with Serum Reproductive Hormones and Ovulation among Healthy Women. J. Nutr..

[14] Kramer M.M., Harman M.T., Brill A.K., Takami M., Preston S.L., Toyloy V.A., Behrman H.R. (1933). Disturbances of reproduction and ovarian changes in the guinea-pig in relation to vitamin C deficiency. Am. J. Physiol. Leg. Content.

[15] Morris G.E. (1953). Hypermenorrhea due to scurvy. Postgrad. Med..

[16] Jacob R.A., Planalto F.S., Agee R.E. (1992). Cellular ascorbate depletion in healthy men. J. Nutr..

[17] Zhou X., Shi H., Zhu S., Wang H., Sun S. (2022). Effects of vitamin E and vitamin C on male infertility: A meta-analysis. Int. Urol. Nephrol..

[18] Asadi N., Bahmani M., Kheradmand A., Rafieian-Kopaei M. (2017). The Impact of Oxidative Stress on Testicular Function and the Role of Antioxidants in Improving it: A Review. J. Clin. Diagn. Res..

[19] Barati E., Nikzad H., Karimian M. (2020). Oxidative stress and male infertility: Current knowledge of pathophysiology and role of antioxidant therapy in disease management. Cell. Mol. Life Sci..

[20] Akmal M., Qadri J., Al-Waili N.S., Thangal S., Haq A., Saloom K.Y., Deruelle F., Baron B., Vani K., Kurakula M. (2006). Improvement in human semen quality after oral supplementation of vitamin C. J. Med. Food.

[21] De Oliveira A.M., Rondo P.H., Barros S.B. (2004). Concentrations of ascorbic acid in the plasma of pregnant smokers and nonsmokers and their newborns. Int. J. Vitam. Nutr. Res..

[22] Scaife A.R., McNeill G., Campbell D.M., Martindale S., Devereux G., Seaton A. (2006). Maternal intake of antioxidant vitamins in pregnancy in relation to maternal and fetal plasma levels at delivery. Br. J. Nutr..

[23] Schjoldager J.G., Tveden-Nyborg P., Lykkesfeldt J. (2013). Prolonged maternal vitamin C deficiency overrides preferential fetal ascorbate transport but does not influence perinatal survival in guinea pigs. Br. J. Nutr..

[24] Juhl B., Lauszus F.F., Lykkesfeldt J. (2017). Poor Vitamin C Status Late in Pregnancy Is Associated with Increased Risk of Complications in Type 1 Diabetic Women: A Cross-Sectional Study. Nutrients.

[25] Mikhail M.S., Anyaegbunam A., Garfinkel D., Palan P.R., Basu J., Romney S.L. (1994). Preeclampsia and antioxidant nutrients: Decreased plasma levels of reduced ascorbic acid, alpha-tocopherol, and beta-carotene in women with preeclampsia. Am. J. Obstet. Gynecol..

[26] Siega-Riz A.M., Promislow J.H., Savitz D.A., Thorp J.M., McDonald T. (2003). Vitamin C intake and the risk of preterm delivery. Am. J. Obstet. Gynecol..

[27] Jain S.K., Wise R., Yanamandra K., Dhanireddy R., Bocchini J.A. (2008). The effect of maternal and cord-blood vitamin C, vitamin E and lipid peroxide levels on newborn birth weight. Mol. Cell. Biochem..

[28] Lee B.E., Hong Y.C., Lee K.H., Kim Y.J., Kim W.K., Chang N.S., Park E.A., Park H.S., Hann H.J. (2004). Influence of maternal serum levels of vitamins C and E during the second trimester on birth weight and length. Eur. J. Clin. Nutr..

[29] Jang W., Kim H., Lee B.-E., Chang N. (2018). Maternal fruit and vegetable or vitamin C consumption during pregnancy is associated with fetal growth and infant growth up to 6 months: Results from the Korean Mothers and Children’s Environmental Health (MOCEH) cohort study. Nutr. J..

[30] Rumbold A.R., Crowther C.A., Haslam R.R., Dekker G.A., Robinson J.S., ACTS Study Group (2006). Vitamins C and E and the risks of preeclampsia and perinatal complications. N. Engl. J. Med..

[31] Roberts J.M., Myatt L., Spong C.Y., Thom E.A., Hauth J.C., Leveno K.J., Pearson G.D., Wapner R.J., Varner M., Thorp J.M. (2010). Vitamins C and E to prevent complications of pregnancy-associated hypertension. N. Engl. J. Med..

[32] Chappell L.C., Seed P.T., Briley A.L., Kelly F.J., Lee R., Hunt B.J., Parmar K., Bewley S.J., Shennan A.H., Steer P.J. (1999). Effect of antioxidants on the occurrence of pre-eclampsia in women at increased risk: A randomised trial. Lancet.

[33] Chappell L.C., Seed P.T., Cstat, Kelly F.J., Briley A., Hunt B.J., Charnock-Jones D., Mallet A., Poston L. (2002). Vitamin C and E supplementation in women at risk of preeclampsia is associated with changes in indices of oxidative stress and placental function. Am. J. Obstet. Gynecol..

[34] Kilkenny C., Browne W.J., Cuthill I.C., Emerson M., Altman D.G. (2012). Improving bioscience research reporting: The ARRIVE guidelines for reporting animal research. Osteoarthr. Cartil..

[35] Tveden-Nyborg P., Johansen L.K., Raida Z., Villumsen C.K., Larsen J.O., Lykkesfeldt J. (2009). Vitamin C deficiency in early postnatal life impairs spatial memory and reduces the number of hippocampal neurons in guinea pigs. Am. J. Clin. Nutr..

[36] Hasselholt S., Tveden-Nyborg P., Lykkesfeldt J. (2015). Distribution of vitamin C is tissue specific with early saturation of the brain and adrenal glands following differential oral dose regimens in guinea pigs. Br. J. Nutr..

[37] Tveden-Nyborg P., Hasselholt S., Miyashita N., Moos T., Poulsen H.E., Lykkesfeldt J. (2012). Chronic vitamin C deficiency does not accelerate oxidative stress in ageing brains of guinea pigs. Basic Clin. Pharmacol. Toxicol..

[38] Lykkesfeldt J., Trueba G.P., Poulsen H.E., Christen S. (2007). Vitamin C deficiency in weanling guinea pigs: Differential expression of oxidative stress and DNA repair in liver and brain. Br. J. Nutr..

[39] Schjoldager J.G., Paidi M.D., Lindblad M.M., Birck M.M., Kjærgaard A.B., Dantzer V., Lykkesfeldt J., Tveden-Nyborg P. (2015). Maternal vitamin C deficiency during pregnancy results in transient fetal and placental growth retardation in guinea pigs. Eur. J. Nutr..

[40] Tveden-Nyborg P., Vogt L., Schjoldager J.G., Jeannet N., Hasselholt S., Paidi M.D., Christen S., Lykkesfeldt J. (2012). Maternal vitamin C deficiency during pregnancy persistently impairs hippocampal neurogenesis in offspring of guinea pigs. PLoS ONE.

[41] Clarke G.L., Allen A.M., Small J.D., Lock A. (1980). Subclinical scurvy in the guinea pig. Vet. Pathol..

[42] Wilson R.L., Lampe K., Matushewski B.J., Regnault T.R.H., Jones H.N. (2021). Time Mating Guinea Pigs by Monitoring Changes to the Vaginal Membrane throughout the Estrus Cycle and with Ultrasound Confirmation. Methods Protoc..

[43] Pullar J.M., Bayer S., Carr A.C. (2018). Appropriate Handling, Processing and Analysis of Blood Samples is Essential to Avoid Oxidation of Vitamin C to Dehydroascorbic Acid. Antioxidants.

[44] Carr A.C., Pullar J.M., Moran S., Vissers M.C.M. (2012). Bioavailability of vitamin C from kiwifruit in non-smoking males: Determination of ‘healthy’ and ‘optimal’ intakes. J. Nutr. Sci..

[45] Smith-Díaz C.C., Magon N.J., McKenzie J.L., Hampton M.B., Vissers M.C.M., Das A.B. (2021). Ascorbate Inhibits Proliferation and Promotes Myeloid Differentiation in TP53-Mutant Leukemia. Front. Oncol..

[46] Festing M.F.W., Altman D.G. (2002). Guidelines for the design and statistical analysis of experiments using laboratory animals. ILAR J..

[47] Shaw J.C., Crombie G.K., Palliser H.K., Hirst J.J. (2021). Impaired Oligodendrocyte Development Following Preterm Birth: Promoting GABAergic Action to Improve Outcomes. Front. Pediatr..

[48] Morrison J.L., Botting K.J., Darby J.R.T., David A.L., Dyson R.M., Gatford K.L., Gray C., Herrera E.A., Hirst J.J., Kim B. (2018). Guinea pig models for translation of the developmental origins of health and disease hypothesis into the clinic. J. Physiol..

[49] Chinoy N.J., Mehta R.R., Seethalakshmi L., Sharma J.D., Chinoy M.R. (1986). Effects of vitamin C deficiency on physiology of male reproductive organs of guinea pigs. Int. J. Fertil..

[50] Bessesen D. (1923). Changes in organ weights of the guinea pig during experimental scurvy. Am. J. Physiol. Leg. Content.

[51] Hamidian S., Talebi A.R., Fesahat F., Bayat M., Mirjalili A.M., Ashrafzadeh H.R., Rajabi M., Montazeri F., Babaei S. (2020). The effect of vitamin C on the gene expression profile of sperm protamines in the male partners of couples with recurrent pregnancy loss: A randomized clinical trial. Clin. Exp. Reprod. Med..

[52] Rafiee B., Morowvat M.H., Rahimi-Ghalati N. (2016). Comparing the Effectiveness of Dietary Vitamin C and Exercise Interventions on Fertility Parameters in Normal Obese Men. Urol. J..

[53] Coker S.J., Smith-Díaz C.C., Dyson R.M., Vissers M.C.M., Berry M.J. (2022). The Epigenetic Role of Vitamin C in Neurodevelopment. Int. J. Mol. Sci..

[54] Blaschke K., Ebata K., Karimi M.M., Zepeda-Martínez J.A., Goyal P., Mahapatra S., Tam A., Laird D.J., Hirst M., Rao A. (2013). Vitamin C induces Tet-dependent DNA demethylation and a blastocyst-like state in ES cells. Nature.

[55] Yin R., Mao S.-Q., Zhao B., Chong Z., Yang Y., Zhao C., Zhang D., Huang H., Gao J., Li Z. (2013). Ascorbic acid enhances Tet-mediated 5-methylcytosine oxidation and promotes DNA demethylation in mammals. J. Am. Chem. Soc..

[56] Ni K., Dansranjavin T., Rogenhofer N., Oeztuerk N., Deuker J., Bergmann M., Schuppe H.-C., Wagenlehner F., Weidner W., Steger K. (2016). TET enzymes are successively expressed during human spermatogenesis and their expression level is pivotal for male fertility. Hum. Reprod..

[57] Vander Borght M., Wyns C. (2018). Fertility and infertility: Definition and epidemiology. Clin. Biochem..

[58] Sadeghi H.M., Adeli I., Calina D., Docea A.O., Mousavi T., Daniali M., Nikfar S., Tsatsakis A., Abdollahi M. (2022). Polycystic Ovary Syndrome: A Comprehensive Review of Pathogenesis, Management, and Drug Repurposing. Int. J. Mol. Sci..

[59] Igarashi M. (1977). Augmentative effect of ascorbic acid upon induction of human ovulation in clomiphene-ineffective anovulatory women. Int. J. Fertil..

[60] Habibzadeh N., Schorah C.J., Smithells R.W. (1986). The effects of maternal folic acid and vitamin C nutrition in early pregnancy on reproductive performance in the guinea-pig. Br. J. Nutr..

[61] Alves C., Rapp A. (2023). Spontaneous Abortion.

[62] McCollin A., Swann R.L., Summers M.C., Handyside A.H., Ottolini C.S. (2020). Abnormal cleavage and developmental arrest of human preimplantation embryos in vitro. Eur. J. Med. Genet..

[63] Gu T.-P., Guo F., Yang H., Wu H.-P., Xu G.-F., Liu W., Xie Z.-G., Shi L., He X., Jin S.-G. (2011). The role of Tet3 DNA dioxygenase in epigenetic reprogramming by oocytes. Nature.

[64] Khoueiry R., Sohni A., Thienpont B., Luo X., Velde J.V., Bartoccetti M., Boeckx B., Zwijsen A., Rao A., Lambrechts D. (2017). Lineage-specific functions of TET1 in the postimplantation mouse embryo. Nat. Genet..

[65] Chu M., Yao F., Xi G., Yang J., Zhang Z., Yang Q., Tian J., An L. (2021). Vitamin C Rescues in vitro Embryonic Development by Correcting Impaired Active DNA Demethylation. Front. Cell Dev. Biol..

[66] Chronopoulou E., Harper J.C. (2015). IVF culture media: Past, present and future. Hum. Reprod. Update.

[67] DiTroia S.P., Percharde M., Guerquin M.-J., Wall E., Collignon E., Ebata K.T., Mesh K., Mahesula S., Agathocleous M., Laird D.J. (2019). Maternal vitamin C regulates reprogramming of DNA methylation and germline development. Nature.

[68] Kawahori K., Kondo Y., Yuan X., Kawasaki Y., Hanzawa N., Tsujimoto K., Wada F., Kohda T., Ishigami A., Yamada T. (2020). Ascorbic acid during the suckling period is required for proper DNA demethylation in the liver. Sci. Rep..

[69] Monsen E.R. (2000). Dietary reference intakes for the antioxidant nutrients: Vitamin C, vitamin E, selenium, and carotenoids. J. Am. Diet. Assoc..

[70] Dror D.K., Allen L.H. (2018). Overview of Nutrients in Human Milk. Adv. Nutr..

[71] Ahmed L., Islam S., Khan N., Nahid S. (2004). Vitamin C content in human milk (colostrum, transitional and mature) and serum of a sample of bangladeshi mothers. Malays. J. Nutr..

[72] Bakshi S., Paswan V.K., Yadav S.P., Bhinchhar B.K., Kharkwal S., Rose H., Kanetkar P., Kumar V., Al-Zamani Z.A.S., Bunkar D.S. (2023). A comprehensive review on infant formula: Nutritional and functional constituents, recent trends in processing and its impact on infants’ gut microbiota. Front. Nutr..

[73] Francis J., Rogers K., Brewer P., Dickton D., Pardini R. (2008). Comparative analysis of ascorbic acid in human milk and infant formula using varied milk delivery systems. Int. Breastfeed. J..

[74] Karra M.V., Udipi S., Kirksey A., Roepke J.L.B. (1986). Changes in specific nutrients in breast milk during extended lactation. Am. J. Clin. Nutr..

[75] Bates C., Prentice A., Paul A., Whitehead R. (1982). Seasonal variations in ascorbic acid status and breast milk ascorbic acid levels in rural Gambian women in relation to dietary intake. Trans. R. Soc. Trop. Med. Hyg..

[76] Tawfeek H.I., Muhyaddin O.M., Al-Sanwi H.I., Al-Baety N. (2002). Effect of maternal dietary vitamin C intake on the level of vitamin C in breastmilk among nursing mothers in Baghdad, Iraq. Food Nutr. Bull..

[77] Schleicher R.L., Carroll M.D., Ford E.S., Lacher D.A. (2009). Serum vitamin C and the prevalence of vitamin C deficiency in the United States: 2003–2004 National Health and Nutrition Examination Survey (NHANES). Am. J. Clin. Nutr..

[78] Carr A.C., Frei B. (1999). Toward a new recommended dietary allowance for vitamin C based on antioxidant and health effects in humans. Am. J. Clin. Nutr..

[79] Carr A.C., Lykkesfeldt J. (2023). Factors Affecting the Vitamin C Dose-Concentration Relationship: Implications for Global Vitamin C Dietary Recommendations. Nutrients.

[80] Carr A.C., Lykkesfeldt J. (2021). Discrepancies in global vitamin C recommendations: A review of RDA criteria and underlying health perspectives. Crit. Rev. Food Sci. Nutr..

