# Supplementary material for: Effects of Low Vitamin C Intake on Fertility Parameters and Pregnancy Outcomes in Guinea Pigs
Source: Nutrients. 2023 Sep 22;15(19):4107. doi: 10.3390/nu15194107 (PMC10574174; doi:10.3390/nu15194107)
Supplement: Supplementary file 1 [file nutrients-15-04107-s001.zip › nutrients-2589255-supplementary.pdf]

nutrients

Supplementary Materials

Effects of Low Vitamin C Intake on Fertility Parameters and Pregnancy Outcomes in Guinea Pigs

Sharna J. Coker [1], Rebecca M. Dyson [1], Carlos C. Smith-Díaz [2], Margreet C. M. Vissers [2,*] and Mary J. Berry [1,*]

Supplementary Materials:

Optimal Vitamin C Diet Ingredients

Specialty Feeds

3150 Great Eastern Hwy
Glen Forrest
Western Australia 6071
p: +61 8 9298 8111
F: +61 8 9298 8700
Email: info@specialtyfeeds.com

Diet SF20-036 — Guinea Pig and Rabbit Diet plus 900mg Vitamin C

A fixed formulation diet based on our Guinea Pig and Rabbit pellets with an additional 900 mg/Kg Vitamin C.

- If Diet is to be autoclaved, the recommended conditions are. Autoclave at 120° C for 20 minutes with a post autoclaving vacuum drying cycle. Some clumping of the diet can be expected, but the diet clumps can usually be easily broken. Modifying the drying time to leave some residual moisture in the diet can minimise the clumping. Do not autoclave at 135° C as this will result in significant clumping that will be difficult to break. It must be remembered that cholesterol is likely to be degraded by autoclaving.

Calculated Nutritional Parameters	
Protein	17.0%
Total Fat	4.2%
Crude Fibre	14.4%
AD Fibre	18.0%
Digestible Energy	11.1 MJ / Kg

Diet Form and Features

- Cereal grain base diet. 4 mm diameter pellets.
- Pack size 4.5 Kg , vacuum packed in oxygen impermeable plastic bags, under nitrogen. Bags are packed into cardboard cartons to protect them during transit. Smaller pack quantity on request.
- Diet suitable for irradiation and for autoclave.
- Lead time 2 weeks for non-irradiation or 4 weeks for irradiation.

Ingredients	
Specialty Feeds Modified Guinea Pig and Rabbit Diet No added C	997.4 g/Kg
Stabilised Vitamin C (Stay C) 35%	2.6 g/Kg

Feeding Recommendations

Feed ad-lib to animals of all ages.

Added Trace Minerals as Fed	
Iron	40 mg/Kg
Copper	13 mg/Kg
Iodine	1.7 mg/Kg
Manganese	90 mg/Kg
Cobalt	0.7 mg/Kg
Zinc	60 mg/Kg
Selenium	0.1 mg/Kg

Added Vitamins as Fed	
Vitamin A (Retinol)	16 000 IU/Kg
Vitamin E (a Tocopherol acetate)	25 mg/Kg
Vitamin K (Menadione)	1.3 mg/Kg
Vitamin B1 (Thiamine)	4 mg/Kg
Vitamin B2 (Riboflavin)	2.7 mg/Kg
Niacin (Nicotinic acid)	20 mg/Kg
Vitamin B6 (Pryridoxine)	2.7 mg/Kg
Calcium Pantothenate	10 mg/Kg
Vitamin B12 (Cyancobalamin)	6.7 ug/Kg
Vitamin C (Ascorbic acid)	900 mg/Kg

Calculated Amino Acids as Fed	
Valine	0.77%
Leucine	1.23%
Isoleucine	0.69%
Threonine	0.62%
Methionine	0.17%
Cysteine	0.25%
Lysine	0.84%
Phenylalanine	0.77%
Tyrosine	0.59%
Tryptophan	0.20%
Arginine	1.36%
Histidine	0.44%

Calculated Total Minerals as Fed	
Calcium	1.11%
Phosphorous	0.72%
Magnesium	0.30%
Sodium	0.20%
Chloride	0.46%
Potassium	1.11%
Sulphur	0.18%
Iron	356 mg/Kg
Copper	23 mg/Kg
Iodine	1.8 mg/Kg
Manganese	126 mg/Kg
Cobalt	0.7 mg/Kg
Zinc	95 mg/Kg
Molybdenum	1.0 mg/Kg
Selenium	0.3 mg/Kg
Cadmium	Trace
Chromium	No data

Calculated Total Vitamins as Fed	
Vitamin A (Retinol)	48 510 IU/Kg
Vitamin D (Cholecalciferol)	No data
Vitamin E (a Tocopherol acetate)	60 mg/Kg
Vitamin K (Menadione)	3.3 mg/Kg
Vitamin C (Ascorbic acid)	900 mg/Kg
Vitamin B1 (Thiamine)	5.6 mg/Kg
Vitamin B2 (Riboflavin)	6.6 mg/Kg
Niacin (Nicotinic acid)	56 mg/Kg
Vitamin B6 (Pryridoxine)	6 mg/Kg
Pantothenic Acid	19 mg/Kg
Biotin	140 ug/Kg
Folic Acid	0.6 mg/Kg
Inositol	No data
Vitamin B12 (Cyancobalamin)	7.3 ug/Kg
Choline	2 270 mg/Kg

Calculated Fatty Acid Composition as Fed	
Myristic Acid 14:0	0.01%
Palmitic Acid 16:0	0.34%
Stearic Acid 18:0	0.15%
Palmitoleic Acid 16:1	0.02%
Oleic Acid 18:1	1.40%
Gadoleic Acid 20:1	0.02%
Linoleic Acid 18:2 n6	0.82%
a Linolenic Acid 18:3 n3	0.15%
Arachadonic Acid 20:4 n6	No data
EPA 20:5 n3	No data
DHA 22:6 n3	No data
Total n3	0.15%
Total n6	0.82%

Calculated data uses information from typical raw material composition. It could be expected that individual batches of diet will vary from this figure. **Diet post treatment by irradiation or autoclave could change these parameters.**

We are happy to provide full calculated nutritional information for all of our products, however we would like to emphasise that these diets have been specifically designed for manufacture by Specialty Feeds.

Low Vitamin C Diet Ingredients

Specialty Feeds
3150 Great Eastern Hwy
Glen Forrest
Western Australia 6071
p: +61 8 9298 8111
F: +61 8 9298 8700
Email: info@specialtyfeeds.com

Diet	
SF20-035	Guinea Pig and Rabbit Diet plus 100mg Vitamin C

A fixed formulation diet based on our Guinea Pig and Rabbit pellets with an additional 100 mg/Kg Vitamin C.

- If Diet is to be autoclaved, the recommended conditions are. Autoclave at 120° C for 20 minutes with a post autoclaving vacuum drying cycle. Some clumping of the diet can be expected, but the diet clumps can usually be easily broken. Modifying the drying time to leave some residual moisture in the diet can minimise the clumping. Do not autoclave at 135° C as this will result in significant clumping that will be difficult to break. It must be remembered that cholesterol is likely to be degraded by autoclaving.

Calculated Nutritional Parameters	
Protein	17.1%
Total Fat	4.2%
Crude Fibre	14.5%
AD Fibre	18.0%
Digestible Energy	11.1 MJ / Kg

Ingredients	
Specialty Feeds Modified Guinea Pig and Rabbit Diet No added C	999.7 g/Kg
Stabilised Vitamin C (Stay C) 35%	0.3 g/Kg

Feeding Recommendations

Feed ad-lib to animals of all ages.

Diet Form and Features

- Cereal grain base diet. 4 mm diameter pellets.
- Pack size 4.5 Kg , vacuum packed in oxygen impermeable plastic bags, under nitrogen. Bags are packed into cardboard cartons to protect them during transit. Smaller pack quantity on request.
- Diet suitable for irradiation and for autoclave.
- Lead time 2 weeks for non-irradiation or 4 weeks for irradiation.

Added Trace Minerals as Fed	
Iron	40 mg/Kg
Copper	13 mg/Kg
Iodine	1.7 mg/Kg
Manganese	90 mg/Kg
Cobalt	0.7 mg/Kg
Zinc	60 mg/Kg
Selenium	0.1 mg/Kg

Added Vitamins as Fed	
Vitamin A (Retinol)	16 000 IU/Kg
Vitamin E (a Tocopherol acetate)	25 mg/Kg
Vitamin K (Menadione)	1.3 mg/Kg
Vitamin B1 (Thiamine)	4 mg/Kg
Vitamin B2 (Riboflavin)	2.7 mg/Kg
Niacin (Nicotinic acid)	20 mg/Kg
Vitamin B6 (Pryridoxine)	2.7 mg/Kg
Calcium Pantothenate	10 mg/Kg
Vitamin B12 (Cyancobalamin)	6.7 ug/Kg
Vitamin C (Ascorbic acid)	900 mg/Kg

Calculated Amino Acids as Fed	
Valine	0.77%
Leucine	1.23%
Isoleucine	0.69%
Threonine	0.62%
Methionine	0.17%
Cysteine	0.25%
Lysine	0.84%
Phenylalanine	0.77%
Tyrosine	0.59%
Tryptophan	0.20%
Arginine	1.36%
Histidine	0.44%

Calculated Total Minerals as Fed	
Calcium	1.11%
Phosphorous	0.72%
Magnesium	0.30%
Sodium	0.20%
Chloride	0.46%
Potassium	1.11%
Sulphur	0.18%
Iron	356 mg/Kg
Copper	23 mg/Kg
Iodine	1.8 mg/Kg
Manganese	126 mg/Kg
Cobalt	0.7 mg/Kg
Zinc	95 mg/Kg
Molybdenum	1.0 mg/Kg
Selenium	0.3 mg/Kg
Cadmium	Trace
Chromium	No data

Calculated Total Vitamins as Fed	
Vitamin A (Retinol)	48 510 IU/Kg
Vitamin D (Cholecalciferol)	No data
Vitamin E (a Tocopherol acetate)	60 mg/Kg
Vitamin K (Menadione)	3.3 mg/Kg
Vitamin C (Ascorbic acid)	900 mg/Kg
Vitamin B1 (Thiamine)	5.6 mg/Kg
Vitamin B2 (Riboflavin)	6.6 mg/Kg
Niacin (Nicotinic acid)	56 mg/Kg
Vitamin B6 (Pryridoxine)	6 mg/Kg
Pantothenic Acid	19 mg/Kg
Biotin	140 ug/Kg
Folic Acid	0.6 mg/Kg
Inositol	No data
Vitamin B12 (Cyancobalamin)	7.3 ug/Kg
Choline	2 270 mg/Kg

Calculated Fatty Acid Composition as Fed	
Myristic Acid 14:0	0.01%
Palmitic Acid 16:0	0.34%
Stearic Acid 18:0	0.15%
Palmitoleic Acid 16:1	0.02%
Oleic Acid 18:1	1.40%
Gadoleic Acid 20:1	0.02%
Linoleic Acid 18:2 n6	0.82%
a Linolenic Acid 18:3 n3	0.15%
Arachadonic Acid 20:4 n6	No data
EPA 20:5 n3	No data
DHA 22:6 n3	No data
Total n3	0.15%
Total n6	0.82%

Calculated data uses information from typical raw material composition. It could be expected that individual batches of diet will vary from this figure. **Diet post treatment by irradiation or autoclave could change these parameters.**

We are happy to provide full calculated nutritional information for all of our products, however we would like to emphasise that these diets have been specifically designed for manufacture by Specialty Feeds.

ISO 9001 SYSTEM CERTIFICATION SGS FEEDSAFE ACCREDITED FEED MANUFACTURER JAS-ANZ AUSTRALIAN TRUSTED TRADER

Supplementary Figures:

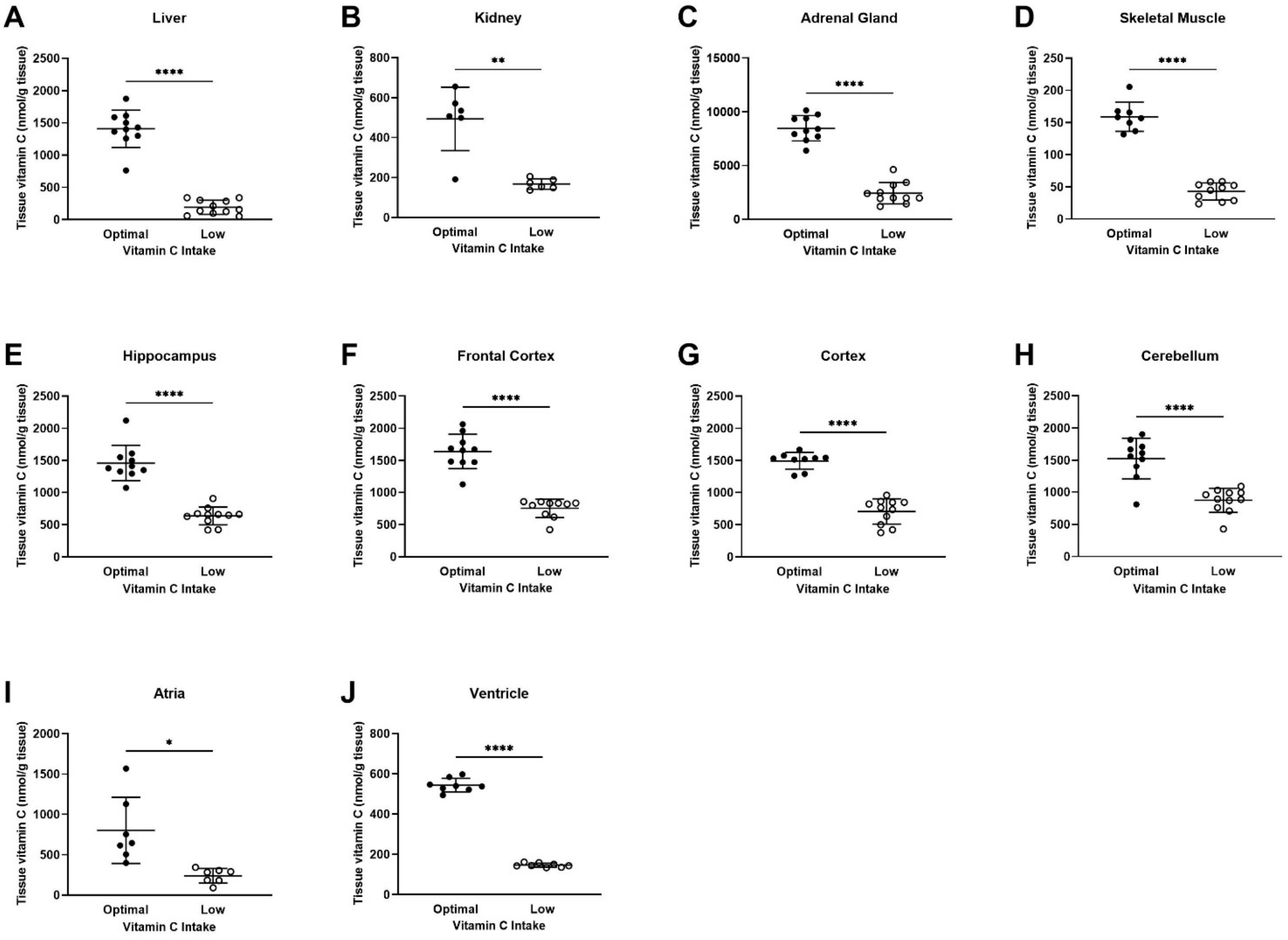

Figure S1. Vitamin C concentrations in the liver (**A**), kidney (**B**), adrenal gland (**C**), skeletal muscle (**D**), hippocampus (**E**), frontal cortex (**F**), cortex (**G**), cerebellum (**H**), atria (**I**), and ventricle (**J**) of adult animals (pooled males and females). Full circles = animals with optimal vitamin C intake and open circles = animals with low vitamin C intake. For liver (n = 10 optimal, n = 11 low), kidney (n = 6 optimal, n = 6 low), adrenal gland (n = 10 optimal, n = 11 low), skeletal muscle (n = 8 optimal, n = 10 low), hippocampus (n = 10 optimal, n = 11 low), frontal cortex (n = 10 optimal, n = 10 low), cortex (n = 9 optimal, n = 11 low), cerebellum (n = 10 optimal, n = 11 low), atria (n = 7 optimal, n = 7 low), and ventricle (n = 8 optimal, n = 8 low). All data is presented as group means ± SD and was analysed using unpaired t-tests (with Welch's correction for heterogeneity of variance applied where appropriate).

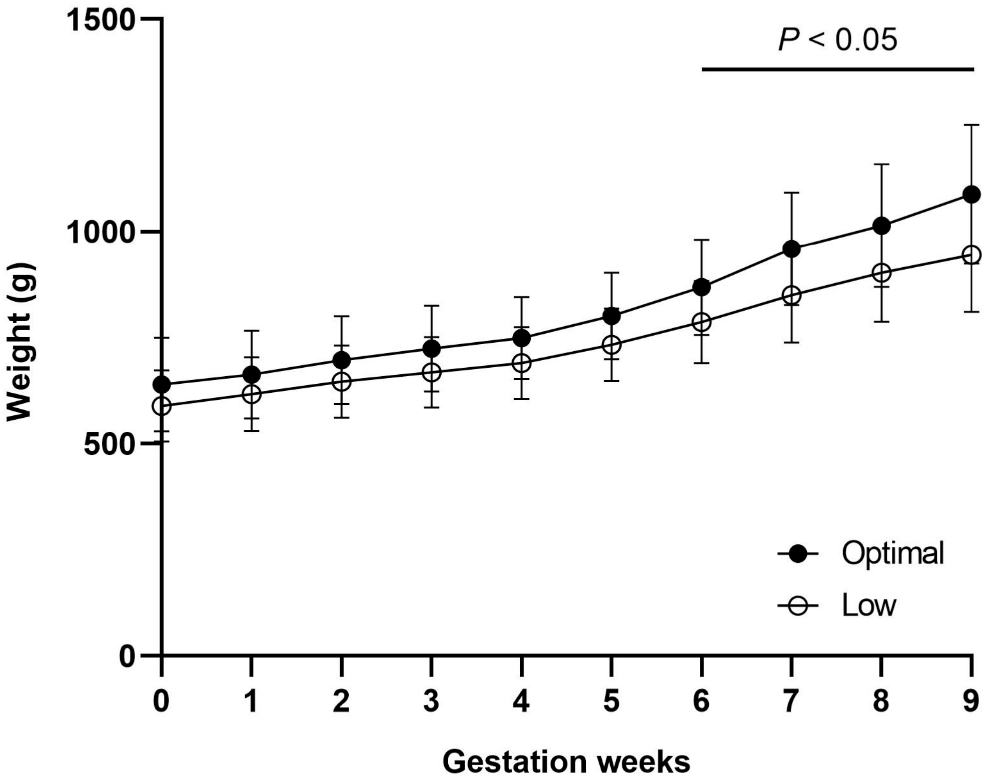

Figure S2. Pregnancy weight gain. The graph represents the average weight gain during pregnancy (day of mating to week of delivery) ± SD. Dams with optimal vitamin C intake (full circles, *n* = 30), and dams with low vitamin C intake (open circles, *n* = 32). Data was analysed using repeated measures mixed-effects ANOVA with diet*time*interaction included as factors.

Supplementary Tables:

Table S1. Adult physical characteristics (raw means and SD).

	Optimal vit. C	(*n*)	Low vit. C	(*n*)	*p*-value
Male weight at enrolment (g)	463.3 ± 86.31	13	489.5 ± 57.92	17	0.3284
Sire weight at mating (g)	744.6 ± 132.4	34	734.9 ± 104.4	36	0.7621
Sire age at mating (weeks)	16.52 ± 3.117	34	17.84 ± 3.601	36	0.1165
Female weight at enrolment (g)	451.9 ± 49.02	35	432.7 ± 48.09	38	0.0959
Dam weight at mating (g)	632.7 ± 111.8	34	588.5 ± 85.86	36	0.0667
Dam age at mating (weeks)	14.93 ± 4.019	34	16.07 ± 5.185	36	0.5020
Testis-to-body weight	0.2282 ± 0.02970	5	0.2171 ± 0.02865	6	0.5443
Ovary-to-body weight	0.01127 ± 0.002767	5	0.008720 ± 0.001536	7	0.0665

Weights for testes and ovaries are expressed as a percentage of body weight (g) on the day of euthanasia.

Table S2. Adult plasma vitamin C concentrations (μM) (adjusted means and SD).

	Optimal vit. C	(*n*)	Low vit. C	(*n*)	*p*-value
Sires at mating	52.15 ± 13.31	10	6.421 ± 2.499	10	< 0.0001*
Dams at mating	35.78 ± 10.86	9	6.243 ± 1.408	7	< 0.0001*
Dams at delivery	36.19 ± 11.14	9	4.805 ± 2.440	8	< 0.0001*

Table S3. Adult tissue vitamin C concentrations (nmol/g tissue) (adjusted means and SD).

	**Optimal vit. C**	**(*n*)**	**Low vit. C**	**(*n*)**	***p*-value**
Testes	2240 ± 334.5	5	655.6 ± 219.9	6	< 0.0001*
Ovaries	2162 ± 719.7	5	476.5 ± 102.1	7	0.0060*
Liver	1408 ± 289.2	10	190.4 ± 109.6	11	< 0.0001*
Kidney	493.6 ± 158.4	6	168.4 ± 25.83	6	0.0037*
Adrenal gland	8466 ± 1180	10	2429 ± 985.2	11	< 0.0001*
Skeletal muscle	158.9 ± 22.69	8	42.91 ± 13.33	10	< 0.0001*
Hippocampus	1460 ± 277.4	10	635.5 ± 137.6	11	< 0.0001*
Frontal cortex	1638 ± 269.1	10	752.7 ± 142.0	10	< 0.0001*
Cortex	1494 ± 134.4	9	705.0 ± 195.5	11	< 0.0001*
Cerebellum	1523 ± 317.1	10	872.0 ± 185.7	11	< 0.0001*
Atria	802.2 ± 409.5	7	239.6 ± 89.92	7	0.0104*
Ventricle	543.7 ± 33.60	8	146.3 ± 9.925	8	< 0.0001*

Table S4. Fertility parameters (raw means and SD).

	**Optimal vit. C**	**Low vit. C**	***p*-value**
Estrous cycle length	15.91 ± 0.7017 (*n* = 35)	15.84 ± 0.7176 (*n* = 38)	0.6130
Unsuccessful matings	30 out of 86 matings = 34.884%	62 out of 120 matings = 51.667%	0.0228*
Failure to establish pregnancy	1 out of 35 females = 2.857%	2 out of 38 females = 5.263%	> 0.9999
Failure to sire progeny	1 out of 13 males = 7.692%	4 out of 17 males = 23.529%	0.3549
Overall reproductive success rate	46 out of 48 animals = 95.833%	49 out of 55 animals = 89.091%	0.2789

Table S5. Pregnancy weight gain (g) (raw means and SD).

	Optimal vit. C (n = 30)	Low vit. C (n = 32)	p-value
Gestation week 0 = day of mating	638.96 ± 110.089	588.794 ± 83.849	0.3985
Gestation week 1	662.887 ± 103.583	616.559 ± 86.763	0.4737
Gestation week 2	696.559 ± 103.179	646.019 ± 85.268	0.3550
Gestation week 3	723.630 ± 100.885	667.869 ± 83.064	0.1944
Gestation week 4	748.630 ± 96.957	689.613 ± 84.812	0.1285
Gestation week 5	800.287 ± 101.701	732.863 ± 85.484	0.0647
Gestation week 6	868.637 ± 112.673	786.025 ± 96.625	0.0305*
Gestation week 7	958.543 ± 132.893	849.766 ± 112.145	0.0099*
Gestation week 8	1014.153 ± 145.014	902.003 ± 115.823	0.0145*
Gestation week 9 = week of delivery	1087.813 ± 163.763	944.772 ± 132.097	0.0042*

Table S6. Pregnancy outcomes (raw means and SD).

	Optimal vit. C	Low vit. C	p-value
Miscarriage (before GA 62)	4 out of 34 pregnancies = 11.765%	2 out of 36 pregnancies = 5.556%	0.4221
Fetal reabsorption	2 out of 34 pregnancies = 5.882%	9 out of 36 pregnancies = 25.000%	0.0463*
Premature delivery (GA 62–66)	0 out of 30 pregnancies = 0.000%	2 out of 34 pregnancies = 5.882%	0.4940
Stillbirth # of pregnancies	5 out of 30 pregnancies = 16.667%	9 out of 34 pregnancies = 26.471%	0.3814
Cumulative adverse pregnancy outcomes	11 out of 34 pregnancies = 32.353%	22 out of 36 pregnancies = 61.111%	0.0188*
Stillbirth # of pups	6 out of 102 pups = 5.882%	11 out of 85 pups = 13.095%	0.1244
Litter size	3.367 ± 0.9994 (n = 30)	2.625 ± 0.9070 (n = 32)	0.0061*
GA of pups at delivery	68.59 ± 1.047 (n = 27)	68.85 ± 1.231 (n = 27)	0.2371
Litter birth weight	95.32 ± 10.41 (n = 27)	97.16 ± 10.01 (n = 27)	0.5119

Table S7. Relative organ weights and body measurements at birth (raw means and SD).

	Male		Female		
	**Optimal vit. C (*n* = 23)**	**Low vit. C (*n* = 19)**	**Optimal vit. C (*n* = 22)**	**Low vit. C (*n* = 18)**	**Model *p*-value**
Body wgt (g)	93.33 ± 14.76	96.25 ± 13.21	91.36 ± 12.87	93.45 ± 14.78	*P* Diet: 0.3228<br>*P* Sex: 0.6329<br>*P* interaction: 0.6068
Brain wgt	2.672 ± 0.4328	2.581 ± 0.2935	2.707 ± 0.4560	2.518 ± 0.2969	*P* Diet: 0.1045<br>*P* Sex: 0.8701<br>*P* interaction: 0.5706
Liver wgt	3.936 ± 0.7353	3.786 ± 0.3614	4.255 ± 0.5623	3.877 ± 0.5245	*P* Diet: 0.0496<br>*P* Sex: 0.1252<br>*P* interaction: 0.3907
Brain-to-liver ratio	0.7073 ± 0.1885	0.6824 ± 0.1132	0.6560 ± 0.1640	0.6678 ± 0.1134	*P* Diet: 0.8520<br>*P* Sex: 0.3513<br>*P* interaction: 0.6042
Heart wgt	0.4148 ± 0.03946	0.4031 ± 0.04820	0.4338 ± 0.04031	0.4030 ± 0.03486	*P* Diet: 0.0197<br>*P* Sex: 0.2807<br>*P* interaction: 0.2751
Kidney wgt	0.4253 ± 0.04484	0.4360 ± 0.03594	0.4338 ± 0.04267	0.4407 ± 0.04137	*P* Diet: 0.3779<br>*P* Sex: 0.4527<br>*P* interaction: 0.7984
Adrenal wgt	0.03094 ± 0.02154	0.02228 ± 0.01041	0.02407 ± 0.01431	0.04991 ± 0.07398	*P* Diet: 0.2210<br>*P* Sex: 0.3100<br>*P* interaction: 0.0536
Testis wgt	0.07463 ± 0.04613	0.07426 ± 0.09005	-	-	*P* Diet: 0.2181
Subcut. Fat wgt	1.463 ± 0.4760	1.397 ± 0.3269	1.342 ± 0.4003	1.297 ± 0.2676	*P* Diet: 0.5261<br>*P* Sex: 0.2089<br>*P* interaction: 0.9063
Visc. Fat wgt	1.059 ± 0.2720	1.040 ± 0.3524	0.7268 ± 0.2826	0.8140 ± 0.1664	*P* Diet: 0.5851<br>*P* Sex: < 0.0001*<br>*P* interaction: 0.3928
	**Male**		**Female**		
	**Optimal vit. C (*n* = 44)**	**Low vit. C (*n* = 36)**	**Optimal vit. C (*n* = 42)**	**Low vit. C (*n* = 35)**	**Model *p*-value**
Crown rump (mm)	128.8 ± 8.819	129.0 ± 8.962	127.7 ± 8.695	126.0 ± 8.674	*P* Diet: 0.6108<br>*P* Sex: 0.1905<br>*P* interaction: 0.5320
Hind limb (mm)	38.48 ± 5.038	35.09 ± 3.675	36.74 ± 4.313	34.65 ± 3.611	*P* Diet: 0.0004*<br>*P* Sex: 0.1452<br>*P* interaction: 0.3882
Hock toe (mm)	38.18 ± 5.108	36.81 ± 3.105	37.32 ± 4.614	35.0 ± 3.55	*P* Diet: 0.0142*<br>*P* Sex: 0.0744<br>*P* interaction: 0.5220
Ponderal index (kg/m^3)	20.16 ± 3.399	22.26 ± 2.923	20.64 ± 3.256	23.24 ± 2.698	*P* Diet: 0.0003*<br>*P* Sex: 0.1907<br>*P* interaction: 0.6328

Weights for organs are expressed as a percentage of body weight (g) on the day of euthanasia. For the liver and heart, the diet p-value of < 0.05 did not remain significant after applying corrections for multiple comparisons.

Table S8. Fractional weight gain (mg/g per day) (adjusted means and SD).

	**Male**			**Female**		
	**Optimal vit. C (n = 21)**	**Low vit. C (n = 17)**	***p*-value**	**Optimal vit. C (n = 20)**	**Low vit. C (n = 17)**	***p*-value**
Day 1	−34.329 ± 30.274	−55.411 ± 24.254	0.1733	−39.781 ± 25.813	−66.150 ± 24.681	0.0339*
Day 2	−9.232 ± 31.049	−19.907 ± 22.397	0.8351	−2.923 ± 26.233	−33.762 ± 19.032	0.0046*
Day 3	8.569 ± 27.223	5.938 ± 21.318	> 0.9999	9.443 ± 26.382	−11.985 ± 17.945	0.0490*
Day 4	20.762 ± 24.696	17.234 ± 21.007	0.9994	22.001 ± 20.558	5.471 ± 17.319	0.0982
Day 5	24.485 ± 23.621	31.138 ± 20.682	0.9842	28.838 ± 24.086	17.773 ± 18.864	0.7938
Day 6	29.240 ± 24.512	28.428 ± 21.425	> 0.9999	28.614 ± 22.219	20.597 ± 21.134	0.9588
Day 7	35.629 ± 13.614	34.082 ± 17.822	> 0.9999	40.321 ± 18.081	24.089 ± 17.221	0.1078

Table S9. Offspring plasma vitamin C concentrations (μM) (adjusted means and SD).

	**Optimal vit. C**	**(*n*)**	**Low vit. C**	**(*n*)**	**p-value**
Day 0	46.78 ± 15.71	8	9.293 ± 5.063	8	0.0002*
Day 7	53.48 ± 15.51	5	60.81 ± 20.84	7	0.5227

Table S10. Salivary cortisol concentrations (ng/mL) (raw means and SD).

	**Optimal vit. C**	**(*n*)**	**Low vit. C**	**(*n*)**	**Model p-value**
Dams	106.2 ± 69.26	10	87.56 ± 62.02	10	0.0924
Offspring	60.62 ± 50.04	20	62.27 ± 38.32	20	
